# C12orf59 Promotes Esophageal Squamous Cell Carcinoma Progression *via* YAP-Mediated Epithelial-Mesenchymal Transition

**DOI:** 10.3389/fonc.2022.927249

**Published:** 2022-07-04

**Authors:** Chunhua Xu, Shan Lin, Yanxin Lu, Longyi Mao, Shi Li, Zesong Li

**Affiliations:** ^1^ Guangdong Provincial Key Laboratory of Systems Biology and Synthetic Biology for Urogenital Tumors, Shenzhen Key Laboratory of Genitourinary Tumor, Department of Urology, The First Affiliated Hospital of Shenzhen University, Shenzhen Second People’s Hospital (Shenzhen Institute of Translational Medicine), Shenzhen, China; ^2^ Guangdong Key Laboratory for Biomedical Measurements and Ultrasound Imaging, School of Biomedical Engineering, Shenzhen University Health Science Center, Shenzhen, China; ^3^ Department of Central Laboratory, Shenzhen Hospital, Beijing University of Chinese Medicine, Shenzhen, China; ^4^ Basic Medical Science Department, Zhuhai Campus of Zunyi Medical University, Zhuhai, China

**Keywords:** C12orf59, esophageal squamous cell carcinoma, the Hippo pathway, YAP, EMT

## Abstract

C12orf59 is a novel gene widely expressed in diverse normal human tissues. Aberrant expression of C12orf59, which is involved in tumor progression, has been reported in a few types of cancer. However, its expression and biological function in esophageal squamous cell carcinoma (ESCC) remain largely unclear. Here, we found that the mRNA and protein levels of C12orf59 were prominently higher in both tumor tissues and most ESCC cell lines. Functionally, C12orf59 overexpression promoted ESCC cell proliferation, migration and invasion, whereas C12orf59 depletion worked oppositely. Mechanistically, C12orf59 exerted its oncogenic function through the induction of epithelial-mesenchymal transition (EMT) of ESCC cells, which relied on Yes-associated protein (YAP) dephosphorylation and nuclear translocation. Constitutively active YAP further facilitated cell migration, invasion and EMT induced by enforced C12orf59 overexpression. On the contrary, increased cell motility and EMT caused by enforced C12orf59 overexpression were dramatically repressed upon YAP inactivation by verteporfin. Thus, we conclude that YAP activation driven by C12orf59 contributes to the malignancy of ESCC through EMT and that targeting drugs for C12orf59 combined with YAP inhibitor may be a potential therapeutic strategy for ESCC.

## Introduction

Esophageal carcinoma (EC), including esophageal squamous cell carcinoma (ESCC) and esophageal adenocarcinoma (EAC), is one of the most lethal malignancies in the world (1). ESCC, the primary type in Southeast Asia and Africa, arises from aberrant proliferation of esophageal squamous epithelium and normally happens in the upper or middle part of the esophagus, which may associate with unhealthy lifestyles, such as thermal injury, alcohol abuse and smoking. EAC, the predominant type in North America and Europe, derives from glandular cells near the stomach and usually occurs in the lower middle of the esophagus, which may relate to gastroesophageal reflux disease, Barrett esophagus and obesity (2, 3). In China, ESCC accounts for approximately 95% of EC cases, and its morbidity and mortality rates rank among the top ten (4). To date, targeted therapy and immunotherapy have been confirmed to play an important role in the treatment of ESCC, but due to questionable efficacy and drug resistance the main therapeutic means are still surgery, radiotherapy and chemotherapy. However, the postoperative survival rate of 5 years is less than quarter (4–6). Recently, genetic alterations have been shown to be ubiquitous in human ESCC samples and play key roles in carcinogenesis through genomic sequencing. Notably, FAT atypical cadherin (FATs) and Ajuba LIM Protein (AJUBA) mutations or Yes-associated protein (YAP) amplification accounted for nearly half of ESCC samples, indicating that dysregulation of the Hippo/YAP signaling could be of great importance for ESCC progression (7–10).

The Hippo pathway, an originally identified kinase cascade in *Drosophila*, is implicated in organ size control and tumor suppression (11, 12). Core components of this pathway consist of Hippo, Salvador (Sav), and Warts, which promote cell apoptosis and restrict proliferation by repressing the downstream effector Yokie in *Drosophila*. The Hippo pathway is evolutionarily conserved in mammals. Mammalian Ste20-like kinases 1/2 (MST1/2), large tumor suppressor 1/2 (LATS1/2) and YAP in mammals are homologs of Hippo, Warts and Yokie in *Drosophila*, respectively. When Hippo signaling is on, MST1/2 and the phosphorylated scaffold protein Sav1 together phosphorylate LATS1/2 and the adaptor protein MOB kinase activator 1 (MOB1). Activated LATS1/2 and MOB1 in turn together phosphorylate the main effectors of the Hippo pathway, YAP and transcriptional coactivator PDZ-binding motif (TAZ), leading to their cytoplasmic sequestration and degradation (13, 14). When Hippo signaling is inactivated, unphosphorylated YAP and TAZ translocate into the nucleus and interact with transcriptional factors, such as TEA-domain family members (TEADs), mothers against DPP homolog (SMADs), runt related transcription factor (RUNX) and zinc finger E-box binding homeobox 1 (ZEB1), thereby regulating genes involved in cell survival, migration and metabolism (15–18). Not surprisingly, substantial evidence has shown that dysregulation of the Hippo pathway is involved in various human cancers. Inactivation of Hippo signaling due to mutations of its upstream inhibitors such as FATs, AJUBA and Dachsous cadherin-related 1 (DCHS1) or its core components such as MST2 and LATS1 (19), and increased expression of its downstream effector YAP has been reported in ESCC (7, 20). However, further study is needed to reveal the molecular mechanism responsible for the regulation of YAP activation in ESCC progression.

The C12orf59 gene (Chromosome 12 open reading frame 59, also termed FLJ31166 or MGC111385), localized on Chromosome 12p13.2, was first cloned in 2002 (21). As a newly identified gene broadly expressed in normal human tissues with especially remarkable expression in kidney, human C12orf59 mRNA was associated with the RNA-binding protein HuR and was predicted to encode transmembrane proteins (22, 23). To date, little is known about the expression pattern and potential function of C12orf59 in human cancers. Previous study reported that C12orf59 was normally undetectable in a panel of genitourinary cancer cell lines, and decreased C12orf59 expression was relevant to poor prognosis and von Hippel-Lindau mutations in renal cell carcinoma (RCC), suggesting C12orf59 as a tumor suppressor in RCC (24). More recently, another study also demonstrated C12orf59 as a tumor suppressor in colon cancer. Briefly, C12orf59 suppression mediated the shedding of extracellular E-cadherin and contributed to activation and internalization of epidermal growth factor receptor (EGFR), resulting in enhanced activation of various downstream signals and subsequent cancer cell survival and invasion. Besides, they also showed that high C12orf59 expression was associated with increased survival in many kinds of cancers, such as lung, liver, breast, and ovarian cancer (25). However, the expression pattern and functional role of C12orf59 in ESCC have not been studied, and it is unclear whether C12orf59 participates in the regulation of the Hippo/YAP signaling in ESCC progression.

Metastasis represents a chief culprit of cancer death in most cancer types. Epithelial-mesenchymal transition (EMT) plays a crucial part in the specific steps of tumor metastasis. Normally, EMT is a common cell biological program that is required during embryonic development at many stages. Activation of EMT makes epithelial cells to lose their apical-basal polarity, cell-cell adhesions, and connection with the basement membrane, and gain stem-cell-like traits, which is featured by high capabilities of migration and invasion (26, 27). Therefore, oncogenic EMT enables tumor cells to disseminate from the primary tumor and colonize at distant organs. It is already known that YAP plays a role in the regulation of ESCC progression through EMT (28), but whether C12orf59 is involved in this process remains unknown. In this study, we found that C12orf59 expression was elevated in ESCC cell lines and tumor tissues. Gain of- and loss of- function studies demonstrated C12orf59 facilitated ESCC cell proliferation, migration and invasion, and induced EMT. C12orf59 worked as an oncoprotein by promoting YAP nuclear translocation and boosting YAP activation. Increased YAP activity was involved in C12orf59-induced increases in ESCC cell motility and EMT. Our results show for the first time that C12orf59 modulates the Hippo/YAP signaling in ESCC progression.

## Materials and Methods

### Cell Culture

Four ESCC cell lines (KYSE140, KYSE180, KYSE450, and KYSE510) and one immortalized esophageal cell line (NE1) were kindly provided by Professor Li Fu (Department of Pharmacology and International Cancer Center, Shenzhen University). The other four ESCC cell lines KYSE30, KYSE150, KYSE410, and TE-1 were purchased from Procell (Procell Life Science&Technology Co,. Ltd, Wuhan, China). The ESCC cell lines were cultured in RPMI-1640 (Gibco, USA) supplemented with fetal bovine serum (Gibco, USA) and 1% penicillin/streptomycin (Gibco, USA). The stable overexpression and knockdown cell lines KYSE30, KYSE410, KYSE510, and TE-1 were cultured in RPMI-1640 supplemented with 10% fetal bovine serum (FBS) and 1 μg/mL puromycin (Selleck Chemicals, USA). NE-1 was cultured in keratinocyte serum-free medium (K-SFM) and EpiLife at a ratio of 1:1 supplemented with 0.05 mg/mL bovine pituitary extract (BPE) and 5 ng/mL human recombinant epidermal growth factor (EGF). For verteporfin treatment, cells were incubated with 2.5 μM (MCE, USA) for KYSE30 in FBS-free RPMI-1640 containing 0.1% BSA for 6 h before cells were harvested. Cells were cultured at 37°C in a humidified atmosphere of 5% CO2. The cell lines were authenticated by short tandem repeat profiling (STR) (Biowing Applied Biotechnology Co. Ltd, Shanghai, China) and to be free of mycoplasma contamination.

### Transfection and Lentiviral Infection

C12orf59 lentiviral expression vector (GV358) and C12orf59 lentiviral shRNA interference vectors (GV493) were constructed by GeneChem (Shanghai, China). C12orf59 specific siRNA sequences (5’-GCAGAAAGCACCTGATCTA-3’ and 5’-GGGTACATCTCTGGTATATAT-3’) were synthesized and subcloned by GeneChem. Constitutively active (CA)-YAP (pCMV-Flag S127A YAP; Addgene plasmid #27370) was a gift from Kunliang Guan. Transfection was performed using Lipofectamine 3000 (Invitrogen, USA) according to the manufacturer’s instruction. Cells transfected with empty vector were used as controls. To generate lentiviruses, expression plasmid (GV358-C12orf59, GV493-shC12orf59#1, or GV493-C12orf59#2) or corresponding control plasmid was co-transfected with Lentiviral Packaging Mix (Sigma, USA) into 293T cells using Lipofectamine 3000, and virus-containing supernatants were harvested and con-centrated at 48 h post-transfection. ESCC cells were transduced with the lentiviruses for 10 h in the presence of polybrene (5 μg/mL) and were subsequently selected with puromycin (1 μg/mL) for a week to establish stable strains.

### RNA Isolation and Real-Time PCR Analyses

Total RNA was extracted using TAKARA MiniBEST Universal RNA Extraction kit according to the manufacturer’s instruction. The primer sequences were shown below: *C12orf59*, forward 5’- CAGCACTCTCCAGAGCACTATCA-3’, reverse 5’-TGGCTACTGTGAAGCGACTCAT-3’; *YAP*, forward 5’-CAAGACCACCTCTTGGCTAG-3’, reverse 5’-CATCTGTTGCTGCTGGTTGG-3’; *ANKRD1*, forward 5’-AGTAGAGGAACTGGTCACTGG-3’, reverse 5’-TGTTTCTCGCTTTTCCACTGTT-3’; *CTGF*, forward 5’-CAGCATGGACGTTCGTCTG-3’, reverse 5’-AACCACGGTTTGGTCCTTGG-3’; *CYR61*, forward 5’-CTCGCCTTAGTCGTCACCC-3’, reverse 5’-CGCCGAAGTTGCATTCCAG-3’; *RPL19*, forward 5’-ATGCCCGAATGCCAGAGAAG-3’, reverse 5’-TCCCCTTCACCTTCAGGTAC-3’. Reverse transcription was performed using TAKARA PrimeScript RT reagent kit. Real-time PCR was performed using Power SYBR Green PCR Master Mix (ABI) on the ABI 7300 Real-Time PCR system (Applied Biosys-tems). Relative gene expression was calculated using the comparative threshold cycle (2^–ΔΔCT^) method. All reactions were performed in triplicate.

### Western Blotting

Cells were lysed in precooled radioimmunoprecipitation assay (RIPA) lysis buffer (Beyotime, China) plus phosphatase and protease inhibitors (Bimake, USA) and soni-cated. Nuclear and Cytoplasmic proteins were extracted using NE-PER Nuclear and Cytoplasmic Extraction Reagents (Thermo Fisher Scientific, USA) according to the manufacturer’s instruction. Total protein concentrations were determined by a Pierce BCA Protein Assay Kit (Thermo Fisher Scientific, USA). Then, 30 μg of denatured protein samples were subjected to SDS-PAGE and transferred to a PVDF membrane (Millipore, USA). Western blotting was performed using anti-C12orf59 (1:1000; NBP2-49272; Novus, USA), anti-β-tubulin (1:4000; ab6046; Abcam, UK), anti-LaminB1 (1:2000; ab16048; Abcam, UK), anti-E-cadherin (1:1000; #3195; Cell Signaling, USA), anti-ZO-1 (1:1000; #8193; Cell Signaling, USA), anti-SNAIL1 (1:1000; #3879; Cell Signaling, USA), anti-Flag (1:2000; #14793; Cell Signaling, USA), anti-Flag (1:2000; F1804; Sigma, USA), anti-phospho-LATS1 (Ser909) (1:1000; #9157; Cell Signaling, USA), anti-LATS1 (1:1000; #3477; Cell Signaling, USA), anti-phospho-MOB1 (Thr35) (1:1000; #8699; Cell Signaling, USA), anti-MOB1 (1:1000; #13730; Cell Signaling, USA), anti-phospho-YAP (Ser127) (1:1000; #4911; Cell Signaling, USA), anti-phospho-YAP (Ser397) (1:1000; #13619; Cell Signaling, USA), or anti-YAP (1:1000; #14074; Cell Signaling, USA) primary antibodies. Corresponding horseradish peroxidase (HRP)-conjugated secondary antibodies were applied, and proteins of interest were detected using an enhanced chemiluminescence (ECL) kit (Millipore, USA) and visualized with the GeneGnome XRQ Chemical Imaging System (Gene Company Limited, Hong Kong, China). β-tubulin was used as loading control for all the western blots.

### Immunohistochemical Staining

A tissue microarray constructed using tumor tissues from 108 patients was pur-chased from Shanghai Outdo Biotech Co. Ltd (HEsoS180Su11; Shanghai, China). Of the 108 patients, 72 had matched adjacent normal tissues. Immunohistochemical staining was performed on the tissue microarray to assess the protein levels of C12orf59. After dewaxing and rehydration, the section was immersed in sodium citrate solution (10 mM, pH 6.0) and heated for antigen retrieval in a microwave oven (high temperature for 8 min and medium low temperature for 10 min), followed by treatment with 3% hydrogen dioxide and incubation in normal goat serum at room temperature for 1 h. The section was then incubated with anti-C12orf59 primary antibody (1:100; 205013-T08; Sino Biological, China) at 4°C overnight. The signals were developed using the UltraSensitive S-P kit (KIT-9710; MXB Biotechnologies, China), and the nuclei were stained with hematoxylin. The IHC score was acquired *via* multiplying the intensity score (0, negative; 1, weak; 2, moderate; 3, strong) by the score for the percentage of positively stained cells (1, ≤25%; 2, 26%-50%; 3, 51%-75%; 4, >75% of positively stained cells). All studies were approved by the Ethics Committee of Shanghai Outdo Biotech Company (Shanghai, China).

### Immunocytochemistry

Cells seeded on glass cover slip were washed with PBS and fixed with 4% para-formaldehyde for 15 min. After permeabilized with 0.5% Triton X-100 for 20 min and blocked with normal goat serum (ab7481; Abcam, UK) for 1 h, the cells were immediately incubated with anti-YAP (1:200; #14074; Cell Signaling, USA) primary antibody at 4 °C overnight. Then, fluorescent secondary antibody (1:800; ab97075; Abcam, UK) was used to incubate cells at room temperature for 1 h. Nuclei were counterstained with DAPI (1:800; D9542; Sigma, USA) for 5 min and mounted with antifade mounting medium (P0126, Beyotime, China). Images were acquired using a confocal fluorescent microscope (ZEISS, Oberkochen, Germany).

### Colony Formation and 5-Ethynyl-2′-Deoxyuridine (EdU) Incorporation Assays

For colony formation assay, cells were seeded into 6-well plates (800 cells/well) and cultured under routine conditions for 10-15 days. Then cell colonies were washed three times with PBS, fixed with 4% paraformaldehyde for 15 min at room temperature, and stained with 0.1% crystal violet (Beyotime, China). The colonies were photographed and counted. To evaluate cell proliferation, cells were seeded into 96-well plates, and 5-ethynyl-2′-deoxyuridine (EdU) incorporation assay was performed. Briefly, cells were incubated with 10 μM EdU for 2 h after reaching a confluency of about 70%, and stained with Apollo^®^ fluorescent dye (Cell-Light EdU Apollo567 *In vitro* Kit, Ribobio, China) according to the manufacturer’s instruction. Images were captured under a fluorescent microscope (Olympus IX73, Baden-Württemberg, Germany) at 567 nm excitation.

### Cell Migration and Invasion Assays

In migration assay, 200 µL of serum-free medium containing 5×10^4^ cells were seeded into the upper chamber of a transwell (8 μm pore; BD Biosciences, USA), and the lower chamber was filled with 750 µL of medium supplemented with 20% FBS as a chemoattractant. In brief, after cell culture for 24 h, the cells remaining on the upper chamber were scraped off with cotton swabs, and the successfully migrated cells were fixed with 4% paraformaldehyde for 15 min, followed by staining with 0.1% crystal violet (Beyotime, China) for 20 min. The invasion assay was similar to that of the migration assay, except that a total of 1×10^5^ cells were seeded into the upper chambers that were precoated with Matrigel (1:8; BD Biosciences, USA). The number of migrated or invaded cells was calculated using five random fields photographed under a light microscope (Nikon DS-Ri2, Tokyo, Japan).

### Statistical Analysis

All analyses were performed using GraphPad Prism 8.0 (GraphPad Software, San Diego, CA, USA). The data were represented as mean ± standard deviation (SD). Statistical differences between two groups were tested using Student’s t-test. Comparisons among three or more groups were conducted using one-way ANOVA with a post-test to correct for multiple comparisons. Chi-square test and Fisher’s exact test were used to analyze the expression frequency of C12orf59 in ESCC tissues and adjacent normal tissues. A P value less than 0.05 was considered statistically significant.

## Results

### C12orf59 Expression Is Elevated in Human ESCC Tissues and Cells

**Figure 1:**
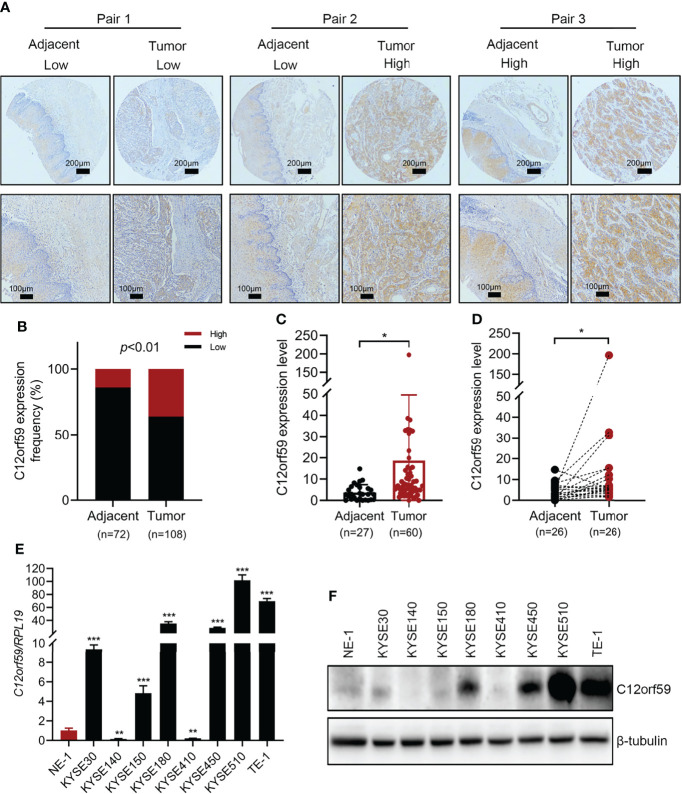
The expression of C12orf59 is increased in ESCC tissues and cell lines. **(A)** Immunohistochemistry for C12orf59 in ESCC and adjacent normal tissues using a tissue microarray at low (upper panel; scale bar, 200 μm) and high magnification (lower panel; scale bar, 100 μm). **(B)** Percentage of ESCC patients with high and low C12orf59 expression in ESCC tissues and adjacent normal tissues. **(C)** Real-time PCR analysis of C12orf59 in ESCC tissues and adjacent normal tissues. **(D)** C12orf59 mRNA levels in 26 paired ESCC tissues and adjacent normal tissues. **(E)** C12orf59 mRNA levels by real-time PCR in immortalized esophageal cell line NE-1 and ESCC cell lines. The relative C12orf59 mRNA levels in ESCC cell lines were normalized with NE-1 cell line. **(F)** C12orf59 protein levels by western blotting in immortalized esophageal cell line NE-1 and ESCC cell lines. *RPL19* was used as the internal control for real-time PCR. β-tubulin was used as the loading control for western blotting. Data were presented as mean values with standard deviations, *p < 0.05; **p < 0.01; ***p < 0.001.

To explore the role of C12orf59 in ESCC, we performed immumohistochemical staining for C12orf59 using a tissue microarray (HEsoS180Su11; Shanghai, China) which contains 108 cancer tissues and 72 matched adjacent normal tissues. We found that C12of59 was highly expressed in ESCC tissues compared with adjacent normal tissues (**Figure 1A**). Based on the expression levels of C12orf59, we divided all ESCC patients into high and low expression groups. Although high C12orf59 expression was not extremely frequent in cancer tissues, there was a significantly higher frequency of up-regulated C12orf59 expression in ESCC tissue (36.1%, 39/108) than that in adjacent normal tissue (13.9%, 10/72) (**Figure 1B**). We also measured the mRNA levels of C12orf59 in a cDNA microarray (cDNA-HEsoS095Su01; Shanghai, China) which contains 67 cancer tissues and 28 matched adjacent normal tissues. Consistently, the mRNA levels of C12orf59 were obviously elevated in ESCC tissues compared with tumor-adjacent tissues (**Figures 1C, D**). Furthermore, the expression of C12orf59 in human ESCC cell lines was also determined by evaluating the mRNA and protein levels of C12orf59 in human immortalized esophageal cells (NE1) and eight ESCC cell lines (KYSE30, KYSE140, KYSE150, KYSE180, KYSE410, KYSE450, KYSE510, and TE-1). We found consistent results between our real-time PCR (**Figure 1E**) and western blotting (**Figure 1F**) analyses revealing that compared with normal esophageal cells, KYSE510 and TE-1 had a dramatically higher expression of C12orf59, and KYSE30, KYSE180 and KYSE450 showed a significantly increased expression of C12orf59. These results demonstrate that C12orf59 was up-regulated in ESCC tissues and cells, indicating an important role of C12orf59 in ESCC progression.

### C12orf59 Enhances ESCC Cell Proliferation

**Figure 2:**
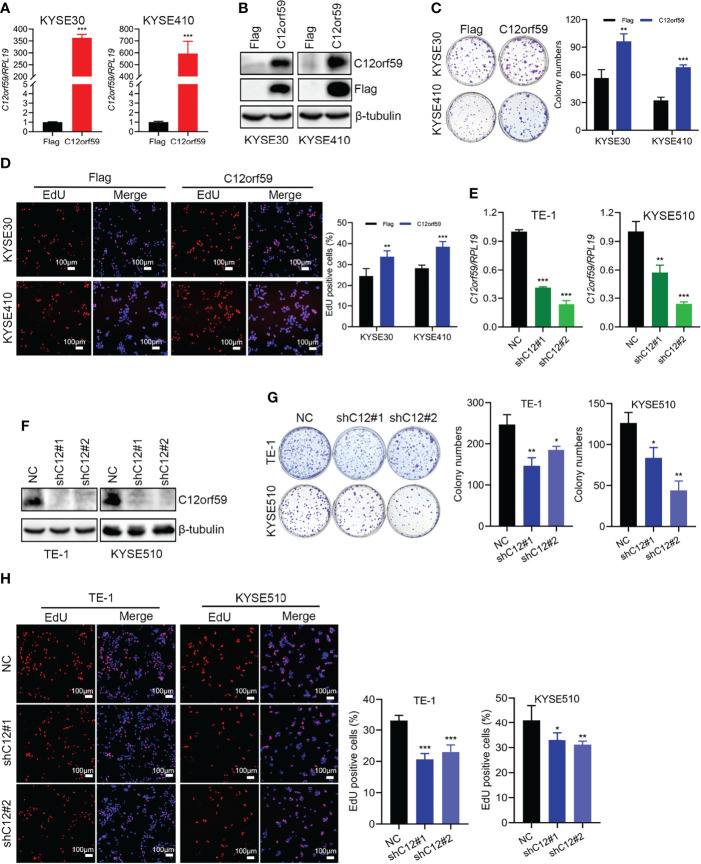
C12orf59 increases the colony formation and proliferation of ESCC cells. **(A, B)** Real-time PCR and western blotting were performed to measure the C12orf59 mRNA and protein levels in KYSE30 and KYSE410 cells with infection of control or C12orf59-expressing lentivirus. **(C)** The capacities of colony formation were evaluated in KYSE30 and KYSE410 cells with infection of control or C12orf59-expressing lentivirus. Colony numbers were counted and quantitative analysis of colony formation was shown. **(D)** EdU incorporation assay was used to measure the cell proliferation of KYSE30 and KYSE410 cells. The relative proliferation rate was quantified by calculating the percentage of EdU-positive cells. Scale bar, 100 μm. **(E, F)** C12orf59 mRNA and protein levels were measured in TE-1 and KYSE510 cells with infection of control or C12orf59-shRNA-expressing lentivirus by real-time PCR and western blotting. **(G)** The capacities of colony formation were evaluated in TE-1 and KYSE510 cells with infection of control or C12orf59-shRNA-expressing lentivirus. Colony numbers were counted and quantitative analysis of colony formation was shown. **(H)** EdU incorporation assay was used to measure the cell proliferation of TE-1 and KYSE510 cells. The relative proliferation rate was quantified by calculating the percentage of EdU-positive cells. Scale bar, 100 μm. *RPL19* was used as the internal control for real-time PCR. β-tubulin was used as the loading control for western blotting. Data were presented as mean values with standard deviations, *p < 0.05; **p < 0.01; ***p < 0.001.

To define the potential role of C12orf59 in ESCC, we performed both gain of- and loss of- function assays in ESCC cell lines to alter C12orf59 expression and studied its role in the regulation of ESCC cell proliferation. The KYSE30 and KYSE410, which displayed a moderate increase and an apparent decrease in C12orf59 levels, while the TE-1 and KYSE510, which exhibited intense increases in C12orf59 levels (**Figures 1E, F**), were subjected to lentiviral infection with either C12orf59-flag overexpression or C12orf59-shRNA interference. The overexpression and knockdown efficiencies were determined by real-time PCR and western blotting. As expected, C12orf59 mRNA and protein levels were significantly increased in KYSE30 and KYSE410 cells (**Figures 2A, B**) and substantially depleted in TE-1 and KYSE510 cells (**Figures 2E, F**). Next, we evaluated the effect of C12orf59 on ESCC cell proliferation using colony formation and 5-ethynyl-2′-deoxyuridine (EdU) incorporation assays. The colony formation assay indicated that C12orf59 overexpression significantly increased the number of colonies by ~41% and ~53% in KYSE30 and KYSE410 cells respectively (**Figure 2C**). By contrast, C12orf59 knockdown induced by shC12orf59#1 or shC12orf59#2 apparently reduced the number of colonies by ~40% and ~25% in TE-1 and ~33% and ~65% in KYSE510 cells (**Figure 2G**). Consistently, high C12orf59 levels contributed to obvious increases in the percentage of EdU positive cells in KYSE30 and KYSE410 cells (**Figure 2D**), but C12orf59 depletion induced significant decreases in the percentage of EdU positive cells in TE-1 and KYSE510 (**Figure 2H**) cells. Taken together, our results suggest that C12orf59 played an important part in the promotion of ESCC cell proliferation.

### C12orf59 Promotes ESCC Cell Migration and Invasion

**Figure 3:**
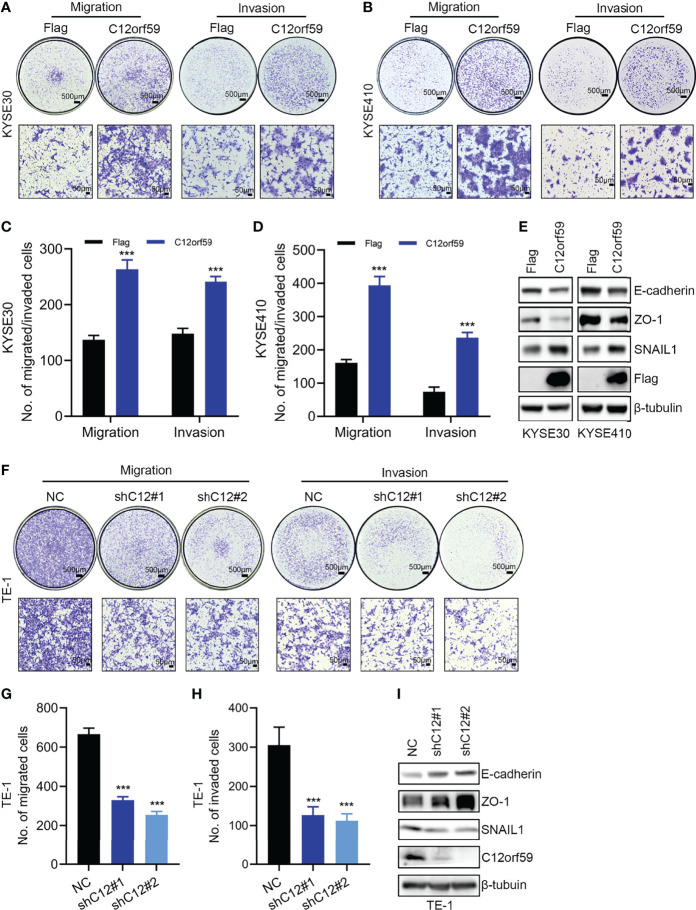
C12orf59 promotes EMT and cellular motility of ESCC cells. **(A, B)** Transwell assays were used to investigate the capabilities of migration and invasion of KYSE30 and KYSE410 cells infected with control or C12orf59-expressing lentivirus. Scale bar, 500 μm for low and 50 μm for high magnification. **(C, D)** Quantitative data of migrated or invaded KYSE30 and KYSE410 cells were shown. **(E)** Protein levels of EMT markers (E-cadherin, ZO-1, and SNAIL1) in KYSE30 and KYSE410 cells with or without C12orf59 overexpression were detected by western blotting. **(F)** Migration and invasion of TE-1 cells infected with control or C12orf59-shRNA-expressing lentivirus were measured by transwell assays. Scale bar, 500 μm for low and 50 μm for high magnification. **(G, H)** Quantitative data of migrated or invaded TE-1 cells were shown. **(I)** Protein levels of EMT markers (E-cadherin, ZO-1, and SNAIL1) and C12orf59 were measured by western blotting in TE-1 cells with or without C12orf59 silencing. β-tubulin was used as the loading control for western blotting. Data were presented as mean values with standard deviations, ***p < 0.001.

Migration and invasion represent the most obvious features of malignant tumors. To explore the oncogenic role of C12orf59 in ESCC, we also studied the effects of C12orf59 on the motility of ESCC cells. We found that enforced overexpression of C12orf59 substantially increased the migratory and invasive capacities of KYSE30 (**Figures 3A, C**) and KYSE410 cells (**Figures 3B, D**). On the other side, suppression of C12orf59 significantly inhibited the ability of TE-1 cells to migrate and invade through transwell membranes (**Figures 3F–H**).

EMT is a developmental process whereby stationary, adherent cells acquire the ability to migrate. However, tumor cells can reactivate EMT program, which increases their aggressiveness. In addition to motility, EMT is associated with enhanced stem cell properties and drug resistance, thus it can drive metastasis, tumor recurrence, and therapy resistance in the context of cancer (26, 29). In this study, we found that C12orf59 overexpression led to reduced levels of the epithelial markers (30) E-cadherin and zonula occludens 1 (ZO-1), and increased levels of the mesenchymal marker (31) SNAIL1 in KYSE30 and KYSE410 cells (**Figure 3E**). On the contrary, E-cadherin and ZO-1 protein levels were up-regulated, but SNAIL1 protein levels were down-regulated in TE-1 cells with C12orf59 abrogation (**Figure 3I**). These results indicate that C12orf59 worked as an oncogenic protein in the acceleration of cell motility through the alteration of EMT in ESCC cells.

### C12orf59 Boosts YAP Activation in ESCC Cells

**Figure 4:**
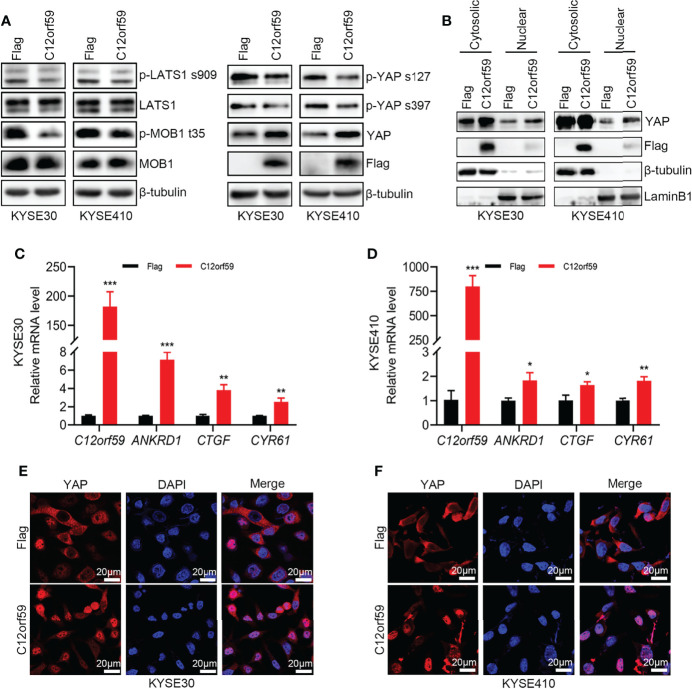
C12orf59 induces YAP nuclear localization. **(A)** Western blotting was used to measure the phosphorylated levels of LATS1 (p-LATS1), MOB1 (p-MOB1) and YAP (p-YAP) in KYSE30 and KYSE410 cells with infection of control or C12orf59-expressing lentivirus. **(B)** Cytosolic and nuclear extracts from KYSE30 or KYSE410 cells with or without C12orf59 overexpression were subjected to western blotting for YAP. β-tubulin was used as the loading control for whole or cytosolic lysates, and LaminB1 was used as the loading control for nuclear extracts. **(C, D)** Expression of C12orf59 and the YAP target genes *ANKRD1*, *CTGF*, and *CYR61* in control and C12orf59-overexpressed KYSE30 and KYSE410 cells were detected by real-time PCR. *RPL19* was used as the internal control for real-time PCR. **(E, F)** Sub-cellular distribution of YAP (red) was examined in control and C12orf59-overexpressed KYSE30 and KYSE410 cells by immunofluorescence. Nuclei were stained with 4′,6-diamidino-2-phenylindole (DAPI; blue). Scale bar, 20 μm. BF, bright field. Data were presented as mean values with standard deviations, *p < 0.05; **p < 0.01; ***p < 0.001.

Previous study showed that C12orf59 was considered as a tumor suppressor by inhibiting downstream signals, including MAPK, PI3K/Akt and Wnt/β-Catenin, induced by EGFR activation in colon cancer (25). However, whether C12orf59 modulates the Hippo pathway, which is of great importance in regulating cell fate, proliferation, apoptosis, and tumorigenesis, mainly by repressing the oncogenic transcription co-activators YAP and TAZ, is unclear. Here, we examined YAP activity in KYSE30 and KYSE410 cells with or without enforced C12orf59 overexpression. The levels of phospho-YAPSer127 and phospho-YAPSer397 were decreased, and total YAP levels were increased in C12orf59-overexpressed KYSE30 and KYSE410 cells compared with their control cells (**Figure 4A**). Of note, cell fractionations showed that more YAP protein was detected in nuclear extracts of KYSE30 and KYSE410 cells with C12orf59 overexpression than in those of control cells (**Figure 4B**). The mRNA levels of YAP target genes *ANKRD1*, *CTGF*, and *CYR61* were higher in both KYSE30 and KYSE410 cells in the presence of enhanced C12or59 expression (**Figures 4C, D**). Furthermore, in contrast to the predominant cytosolic expression in control cells, YAP was mainly localized in the nuclei of KYSE30 and KYSE410 cells when C12orf59 was forcibly introduced (**Figures 4E, F**). To understand the underlying mechanism by which C12orf59 facilitated YAP activation, we also examined phosphorylated LATS1 and MOB1 levels in KYSE30 and KYSE410 cells. We found that both phospho-LATS1Ser909 and phospho-MOB1Thr35 protein levels were significantly decreased when C12orf59 were overexpressed in KYSE30 and KYSE410 cells compared with control cells (**Figure 4A**). LATS1/2 and MOB1 need to function together to control YAP phosphorylation, the decreased LATS1 and MOB1 phosphorylation may explain the reduced phospho-YAP levels in C12or59-introduced ESCC cells. These results signify that C12orf59 stimulated YAP activity possibly *via* modulating upstream signal transduction of the Hippo pathway.

### Reinforced YAP Activity Further Prompts EMT and Motility of ESCC Cells

**Figure 5:**
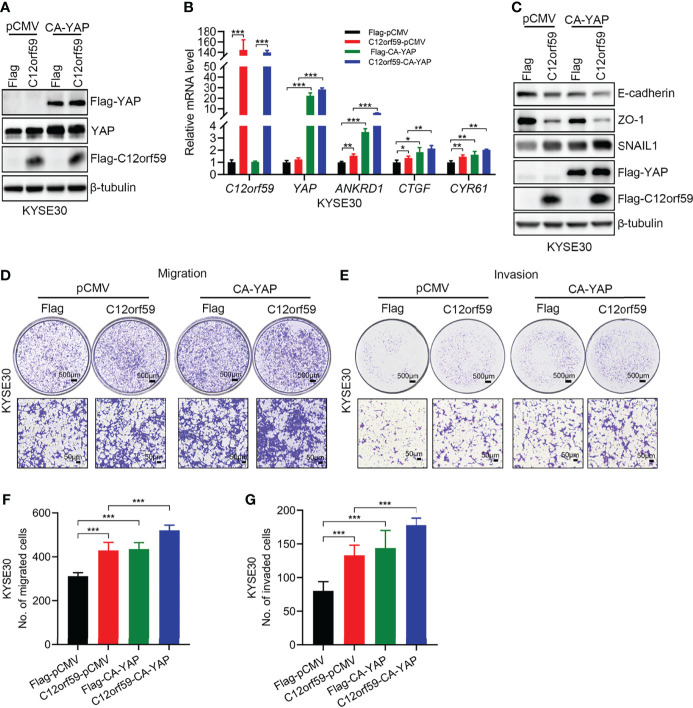
YAP activation facilitates the EMT and cellular motility of ESCC cells. **(A)** KYSE30 cells with or without C12orf59 overexpression were transfected with control or constitutively active YAP (CA-YAP) plasmid. Cell lysates were subjected to western blotting for Flag and YAP to examine transfection efficiency. **(B)** Real-time PCR was performed to examine the mRNA levels of C12orf59, YAP, and YAP target genes (*ANKRD1*, *CTGF*, and *CYR61*) in control and C12orf59-overexpressed KYSE30 cells introduced with or without CA-YAP-expressing plasmid. *RPL19* was used as the internal control for real-time PCR. **(C)** Western blotting was performed to evaluate the protein levels of EMT markers (E-cadherin, ZO-1, and SNAIL1) in control or C12orf59-overexpressed KYSE30 cells transfected with or without CA-YAP. β-tubulin was used as the loading control for western blotting. **(D, E)** The effect of CA-YAP on cellular motility was investigated by transwell assays in control or C12orf59-overexpressed KYSE30 cells introduced with control or CA-YAP-expressing plasmid. Scale bar, 500 μm for low and 50 μm for high magnification. **(F, G)** The number of migrated or invaded cells was counted and quantified. Data were presented as mean values with standard deviations, *p < 0.05; **p < 0.01; ***p < 0.001.

To define whether YAP activation participates in EMT, migration and invasion of ESCC cells, we performed cell transfection to introduce constitutively active YAP (CA-YAP; pCMV-Flag S127A YAP), compelling YAP to be retained in nuclei whereby cells achieve an increased total output of YAP activity, into KYSE30 cells with or without C12orf59 overexpression. We examined the transfection efficiencies by real-time PCR and western blotting, and found that YAP mRNA and protein levels were significantly increased in both C12orf59-overexpressed and control cells (**Figures 5A, B**). Of note, consistent with the above-mentioned results (**Figure 4A**), increased total YAP protein level was always observed in cells bearing enforced C12orf59 overexpression compared with control cells in the absence of CA-YAP (**Figure 5A**). Additionally, the mRNA levels of YAP target genes *ANKRD1*, *CTGF*, and *CYR61* were apparently enhanced upon CA-YAP introduction, suggesting a further heightened YAP activity, regardless of C12orf59 expression compared with their corresponding control cells (**Figure 5B**). Next, we investigated the influence of CA-YAP on EMT and motility of ESCC cells. We found that CA-YAP induced obvious reductions in E-cadherin and ZO-1 protein levels and a distinct increase in SNAIL1 expression in cells with endogenous C12orf59, whereas co-expression of exogenous C12orf59 and CA-YAP further lowered E-cadherin and ZO-1, and further improved SNAIL1 expression compared with only C12orf59-introduced cells (**Figure 5C**), indicating that C12orf59-induced EMT was aggravated by CA-YAP. With regard to cell motility, not only did CA-YAP enhance the migratory and invasive capacities of control cells, it exacerbated the migration and invasion of C12orf59-overexpressed cells (**Figures 5D–G**). Together, YAP activation made a difference in the promotion of EMT and cellular motility of ESCC cells.

### YAP Mediates C12orf59-Driven Malignancy of ESCC Cells

**Figure 6:**
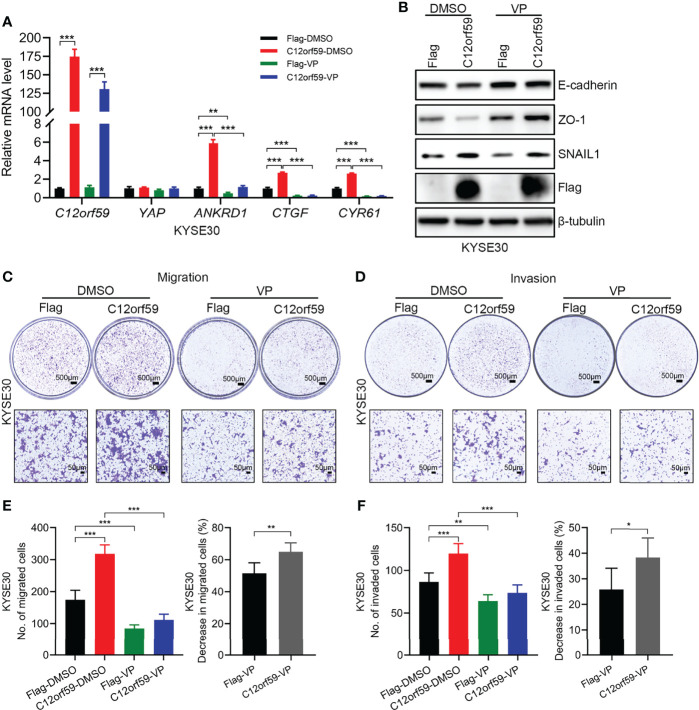
C12orf59 contributes to the EMT and cellular motility of ESCC cells *via* YAP activation. **(A)** Real-time PCR was used to analyze the mRNA levels of C12orf59, YAP, and YAP target genes (*ANKRD1*, *CTGF*, and *CYR61*) in control and C12orf59-overexpressed KYSE30 cells treated with or without verteporfin (YAP inhibitor, 2.5 μM) for 6 h. *RPL19* was used as the internal control for real-time PCR. **(B)** Western blotting was conducted to analyze the protein levels of EMT markers (E-cadherin, ZO-1, and SNAIL1) in control or C12orf59-overexpressed KYSE30 cells in the absence or presence of verteporfin. β-tubulin was used as the loading control for western blotting. **(C, D)** Transwell assays were used to inspect the effect of YAP inhibition by verteporfin on cell migration and invasion in control or C12orf59-overexpressed KYSE30 cells and representative images were shown. Scale bar, 500 μm for low and 50 μm for high magnification. **(E)** The number of migrated cells and the percentage of reduction in cell migration induced by verteporfin was counted, calculated, and quantified. **(F)** The number of invaded cells and the percentage of reduction in cell invasion induced by verteporfin was counted, calculated, and quantified. Data were presented as mean values with standard deviations, *p < 0.05; **p < 0.01; ***p < 0.001.

To define whether the oncogenic functions of C12orf59 relies on increased YAP activity, we used verteporfin (VP), a YAP inhibitor, which restrains YAP activity by destroying YAP-TEADs interaction (32), to treat ESCC cells. In the presence of VP, YAP activity was tremendously abrogated, as illustrated by the significant decreases in the mRNA levels of YAP target genes *ANKRD1*, *CTGF*, and *CYR61* (**Figure 6A**). As with EMT, VP treatment resulted in increased E-cadherin and ZO-1 protein levels, and slightly decreased SNAIL1 protein level in control cells, meaning that YAP inhibition could ameliorate the basal level of EMT. For another, the reduced protein levels of E-cadherin and ZO-1 because of C12orf59 overexpression were significantly restored and even boosted after VP treatment. Besides, compared with DMSO group, SNAIL1 protein level was reduced in C12orf59-overexpressed cells in the combination of VP, although still a little higher than that in control cells (**Figure 6B**). Accordingly, cell migration and invasion were dramatically impeded in the presence of VP in ESCC cells with or without C12or59 overexpression (**Figures 6C, D**). Of note, VP treatment resulted in decreased migration and invasion to a greater extent in C12orf59-overexpressed cells (**Figures 6E, F**). Together, inhibition of YAP activity hindered the intrinsic EMT and motility of ESCC cells, and C12orf59-dependent YAP activation mediated the malignant behaviors of ESCC cells probably through EMT resulting from the aberrant expression of C12orf59.

## Discussion

Recently, evidences have been emerging in understanding the biologic functions of C12orf59 in tumorigenesis, but the role of C12orf59 in the progression of ESCC remained unknown. In this study, we found C12orf59 was frequently overexpressed in ESCC tissues and cell lines, and the oncogenic functions of C12orf59 were reported in cancer cell proliferation, migration and invasion. More importantly, high C12orf59 expression was associated with increased YAP activity, which mediated the malignant behaviors of ESCC cells. We found C12orf59 led to YAP activation by promoting YAP dephosphorylation and nuclear translocation, and increased YAP activity facilitated cancer cell EMT, migration and invasion. Here, we have shown for the first time that C12orf59 is a putative oncogene in ESCC and exerts its oncogenic role by modulating the activity of YAP, which is a well-known oncoprotein recognized as the main downstream effector of the Hippo pathway (33, 34). Therefore, this work is the first to demonstrate a relationship between C12orf59 and the Hippo pathway.

Whether C12orf59 functions as an oncogene or tumor suppressor seems to be cancer type and context dependent. We previously identified C12orf59 as a putative tumor suppressor in RCC, but the molecular mechanism of how C12orf59 insufficiency contributes to the progression of RCC remains obscure. Consistently, it has been recently reported that C12orf59 functioned as a tumor suppressor in human colon carcinoma and exerted its roles by protecting E-cadherin from proteasomal degradation and extracellular shedding (25). On the contrary, C12orf59 expression induced a significant decrease in the protein level of E-cadherin in ESCC cells, thus leading to EMT and increased cellular motility in the present study, indicating that C12orf59 also functions as an oncogene.

Epithelial-mesenchymal transition (EMT) is a developmental program that epithelial cells shed its connections to neighboring cells, and acquire the properties of mesenchymal cells with migratory capacities. Since EMT promotes cellular motility, it is not surprising that considerable progress has been made in revealing the close correlation between EMT and the dissemination of cancer cells (26). Moreover, among the various molecules that regulate EMT, TEAD transcription factors and their transcriptional co-activator YAP, a powerful oncoprotein, are crucial regulators that control the expression of genes critical for EMT and metastasis (27, 35, 36). Additionally, substantial evidences have revealed that the YAP-TEADs functional module plays a positive role in the progression of ESCC (37, 38). On the other hand, YAP was regarded as a candidate oncogene in ESCC decades ago (20). Although many new regulators that play a part in modulating YAP activity have been identified in ESCC, such as RACO-1, PARK2, SHARPIN and SOX9 (39–42). Whether C12orf59 alters YAP activity in ESCC remains unknown. In this study, we found high C12orf59 expression positively correlated with YAP activation, and enhanced YAP activity prompted the EMT, migration and invasion of ESCC cells as indicated by ectopic overexpression of CA-YAP and pharmacological inhibition of intrinsic YAP. As a transcriptional co-activator, YAP activates many transcription factors, such as p73, RUNX, ERBB4, SMADs and TEADs, with TEADs serving as the major YAP-interacting transcription factors (43–45). Here, whether YAP-TEADs functional module responsible for the induction of EMT-related molecules in ESCC cells with differential C12orf59 expression remains to be further investigated.

We also found that along with high C12orf59 expression, the levels of total YAP protein were significantly increased whereas phosphorylated YAP levels were reduced. Since YAP phosphorylation results in the cytoplasmic sequestration and proteasomal degradation of YAP, it is no surprise that an increase in total YAP was observed here. It is unknown by this study how C12orf59 regulates YAP activation, but we demonstrated that the phosphorylated levels of both LATS1 and MOB1, upstream kinases of YAP, were decreased by C12orf59 overexpression, suggesting that C12orf59 may have an passive influence on the signal transduction of Hippo signaling, which leads to YAP dephosphorylation and activation. And as a matter of fact, numerous upstream modulators involved in the Hippo pathway have been identified, such as cell polarity, cell junction factors, mechanotransduction, and G-protein-coupled receptor (GPCR) signaling (46). In this study, cell junction factors E-cadherin and ZO-1 were obviously suppressed by C12orf59. It is possible that C12orf59-induced decreases in E-cadherin and/or ZO-1 could signal to the Hippo pathway, therein regulates YAP activation.

E-cadherin is an important mediator of intercellular contact in epithelial cells. Here, we found that the protein level of E-cadherin was further suppressed by enforced C12orf59 overexpression in the combination of YAP activation, whereas the reduced protein level of E-cadherin resulting from C12orf59 overexpression was abrogated upon YAP inhibition, suggesting YAP inhibits E-cadherin expression in ESCC cells. Importantly, a previous study demonstrated that there is an exquisite correlation between YAP activity and E-cadherin/NF2-regulated ferroptosis. They found accumulated E-cadherin because of increased cell density activates the intracellular NF2 (also known as merlin) and Hippo signaling pathway in HCT116 cells, thereby inhibiting YAP activity and consequent YAP-dependent up-regulation of multiple ferroptosis modulators (47). This study reveals that E-cadherin negatively regulates YAP activity in certain cancer cells. Collectively, it is likely in cancer cells especially those undergoing EMT, there is a negative feedback loop between E-cadherin and YAP activity, but whether this assumption works in ESCC cells in our study remains to be explored. Notably, a recent study reported the identification of the single *Drosophila* ZO-1 protein Polychaetoid (Pyd) acting upstream of the core components in the Hippo pathway during the blue- and green-sensitive photoreceptor subtype binary fate choice (48). Likewise, another integral tight junction protein named claudin 18 (CLDN18) was identified as a regulator of YAP activity that serves to restrict organ size and tumorigenesis. Interestingly, overexpressed CLDN18 associated with ZO-1 and phosphorylated YAP in MLE-15 cell membranes, suggesting that CLDN18 regulation of YAP activity involves phosphorylated-YAP sequestration at tight junctions (TJs) (49). Therefore, C12orf59-induced ZO-1 reduction also might be attributed to increased YAP nuclear translocation, as well as subsequent activated YAP-mediated further suppression of ZO-1 in our study. However, it is unclear by this study how C12orf59 regulates the expression of E-cadherin and ZO-1 in ESCC cells. Nevertheless, in a recent study, it was shown that there is an interaction between C12orf59 and E-cadherin, although this interaction contributes to the stabilization of E-cadherin (25), suggesting that C12orf59 might regulate cell junction factors through direct protein-protein interaction.

In summary, we found that C12orf59 was frequently elevated in ESCC tissues and cell lines and that a high level of C12orf59 expression drove ESCC cell proliferation, EMT, migration and invasion. Meanwhile, knockdown of C12orf59 worked oppositely, suggesting that C12orf59 exerts oncogenic roles in the progression of ESCC. Mechanically, C12orf59 acted as an oncogene in ESCS through increasing the activity of another well-known oncoprotein YAP. High C12orf59 expression suppressed the Hippo pathway and prompted nuclear entry of YAP, leading to the down-regulation of E-cadherin and ZO-1, and enhanced cellular motility. Therefore, C12orf59 is a novel candidate oncogene in ESCC and a newly identified modulator of the Hippo/YAP signaling.

## Data Availability Statement

The original contributions presented in the study are included in the article/supplementary material. Further inquiries can be directed to the corresponding author.

## Ethics Statement

The studies involving human participants were reviewed and approved by the Ethics Committee of Shanghai Outdo Biotech Company (Shanghai, China). Written informed consent for participation was not required for this study in accordance with the national legislation and the institutional requirements.

## Author Contributions

Conceptualization, CX and ZL; methodology, CX, ShaL, YL, LM, and ShiL; formal analysis, CX, ShaL, YL, and ZL; writing-original draft preparation, CX; writing-review and editing, CX and ZL; visualization, CX; supervision, ZL; project administration, ZL; funding acquisition, CX and ZL. All authors have read and agreed to the published version of the manuscript.

## Funding

This research was funded by the National Natural Science Foundation of China (82100706, 81972366), the Postdoctoral Science Foundation of China (2021M692219), the National Science Foundation Projects of Guangdong Province (2022A1515012606), and the Special Project of Science and Technology for Sustainable Development, Shenzhen Municipal Science and Technology Innovation Committee (KCXFZ20211019143336002, JCYJ20200109120208018).

## Conflict of Interest

The authors declare that the research was conducted in the absence of any commercial or financial relationships that could be construed as a potential conflict of interest.

## Publisher’s Note

All claims expressed in this article are solely those of the authors and do not necessarily represent those of their affiliated organizations, or those of the publisher, the editors and the reviewers. Any product that may be evaluated in this article, or claim that may be made by its manufacturer, is not guaranteed or endorsed by the publisher.
